# *GhCNGC13* and *32* Act as Critical Links between Growth and Immunity in Cotton

**DOI:** 10.3390/ijms25010001

**Published:** 2023-12-19

**Authors:** Song Peng, Panyu Li, Tianming Li, Zengyuan Tian, Ruqiang Xu

**Affiliations:** 1 Zhengzhou Research Base, State Key Laboratory of Cotton Biology, Zhengzhou University, Zhengzhou 450001, China; pengsong9509@gmail.com (S.P.); lipy0405@gmail.com (P.L.); ltm678@hotmail.com (T.L.); 2 School of Agricultural Sciences, Zhengzhou University, Zhengzhou 450001, China

**Keywords:** cotton, cyclic nucleotide-gated ion channels (CNGCs), regulation, immunity, growth, photosynthesis

## Abstract

Cyclic nucleotide-gated ion channels (CNGCs) remain poorly studied in crop plants, most of which are polyploid. In allotetraploid Upland cotton (*Gossypium hirsutum*), silencing *GhCNGC13* and *32* impaired plant growth and shoot apical meristem (SAM) development, while triggering plant autoimmunity. Both growth hormones (indole-3-acetic acid and gibberellin) and stress hormones (abscisic acid, salicylic acid, and jasmonate) increased, while leaf photosynthesis decreased. The silenced plants exhibited an enhanced resistance to *Botrytis cinerea*; however, *Verticillium* wilt resistance was weakened, which was associated with *LIPOXYGENASE2 (LOX2)* downregulation. Transcriptomic analysis of silenced plants revealed 4835 differentially expressed genes (DEGs) with functional enrichment in immunity and photosynthesis. These DEGs included a set of transcription factors with significant over-representation in the HSF, NAC, and WRKY families. Moreover, numerous members of the GhCNGC family were identified among the DEGs, which may indicate a coordinated action. Collectively, our results suggested that *GhCNGC13* and *32* functionally link to photosynthesis, plant growth, and plant immunity. We proposed that *GhCNGC13* and *32* play a critical role in the “growth–defense tradeoff” widely observed in crops.

## 1. Introduction

Cyclic nucleotide-gated ion channels (CNGCs) are non-selective channels identified in both animals and plants [1]. Until now, plant CNGCs have been mainly understood from considerably extensive studies in the model plant Arabidopsis over the past two decades. The *Arabidopsis thaliana* genome contains 20 genes encoding CNGC family members, which are phylogenetically classified into four groups (I-IV) and two subgroups (IV-A and IV-B) [2]. These genes are functionally implicated in almost all aspects such as growth, immunity and stress responses, nutrition uptake and transport [3,4]. Thus, plant CNGCs may offer great potential for practical application in crop breeding and improvement. 

Plant CNGCs are typically in the plasma membrane; however, some of them may also be in intracellular organelles, including the mitochondria, chloroplasts, vacuoles, endoplasmic reticulum, and the Golgi apparatus [1]. They mainly mediate Ca^2+^ flux, although conductance for other cations like Na^+^, K^+^, Pb^2+^, and Cd^2+^ has been reported in a few cases [3]. Ca^2+^ is a distinct mineral that functions as both a nutrient and a signal in all eukaryotes [5]. Ca^2+^ signaling is central to both pattern- and effector-triggered plant immunity, with the generation of characteristic cytoplasmic Ca^2+^ in response to various stimuli such as microbes, viruses, herbivores, and parasitic plants [6]. Ca^2+^ also plays multifaceted roles in almost all abiotic stress responses [7]. Intracellular changes in free Ca^2+^ levels act as critical regulators in many growth and developmental processes of plants [8]. Ca^2+^ is an essential plant nutrient required for various structural roles in the cell wall and membranes, and as a counter-cation for inorganic and organic anions in the vacuole [9]. 

All plant CNGCs contain a cyclic nucleotide-binding domain (CNBD) located within the C-terminus [10]. CNBDs can bind cyclic nucleotides [11], and cAMP or cGMP application stimulates CNGC activity [3]. Plant CNGCs also have one or more calmodulin (CaM)-binding domain (CaMBD) as well as a Ca^2+^-independent isoleucine-glutamine (IQ) CaM-binding motif [1,12]. Experimental data have confirmed that CaM directly interacts with CNGCs [13]. Recent advances indicate that plant CNGCs act as a downstream target of phosphorylation in the recognition of pathogen/microbe-associated molecular patterns (PAMPs/MAMPs) [13]. AtCNGC17 interacts with the plasma-membrane-localized H^+^-ATPases AHA1 and AHA2, as well as BAK1, which may form a functional complex with the growth-regulating phytosulfokine (PSK) receptor PSKR1 [14]. Like PSKR1, the BAK1 leucine-rich repeat receptor-like kinase (LRR-RLK) superfamily can associate with various pattern recognition receptors (PRRs) as a coreceptor, forming functional receptor complexes that regulate physiological responses from growth and development to stress responses and immunity [15,16]. Auxin is a key regulator of plant growth and development, and it has been established that AtCNGC14 links Ca^2+^ signaling with auxin growth responses in roots, such as gravitropism and root hair morphogenesis [17,18,19,20]. AtCNGC2 is well known to be involved in plant immunity and Ca^2+^ signaling due to the autoimmune phenotypes exhibited by null mutants, but it also balances cellular auxin perception by influencing auxin biosynthesis [21]. These findings imply that CNGCs may have a more widespread role in regulating plant growth and development through auxin and/or the complex network of hormone signaling. Collectively, CNGCs might act as a converging point of Ca^2+^/CaM, cyclic nucleotides, and phytohormone signaling and thus have a thorough and profound impact on plant life. 

Plants have a much larger family of CNGCs compared to animals, and these genes are differentially expressed in different tissues, being highly responsive to multiple stimuli such as developmental cues, hormones, and biotic and abiotic stresses [22,23,24,25,26,27]. CNGCs form a homo- or hetero-tetramer protein complex producing an ion channel [28,29,30]. Very interestingly, it has been recently found that different CNGCs and CNGC combinations make distinctive contributions to the regulation of channel activities [29]. It will be interesting to investigate the functional diversification of CNGCs in different plants, especially crops that are mostly polyploid species with a functional redundancy among CNGC isoforms and a complexity of CNGC multimers. 

Activation of plant immunity by exogenous signals or mutations has long been associated with what is commonly known as the growth–defense tradeoff [31,32]. This phenomenon also exists in the context of many other abiotic and biotic factors, including light, the circadian clock, CO_2_, temperature, humidity, water, nutrients, and the microbiome [33]. Currently, the molecular mechanisms underlying the growth–defense tradeoff remain elusive. In agriculture settings, crops have often been bred to maximize growth-related traits, which could inadvertently result in the loss of useful genetic traits for biotic defenses [33]. Cotton (*Gossypium* spp.) is an industrial crop cultivated worldwide for its fiber and seed oil, and it is often used as a model system for polyploidization, cell elongation, and cell wall biosynthesis [34]. Upland cotton (*G. hirsutum*) is an allotetraploid species (2n = 4x = 52, AADD) that originated from transoceanic diffusion, natural hybridization, and doubling of the diploid A and D genomes [34]. The present study was intended to characterize the biological functions and regulatory mechanisms of *GhCNGC13* and *32* in Upland cotton. 

## 2. Results and Discussion

### 2.1. GhCNGC13 and 32 Are Essential for Cotton Growth and Development

**Figure 1:**
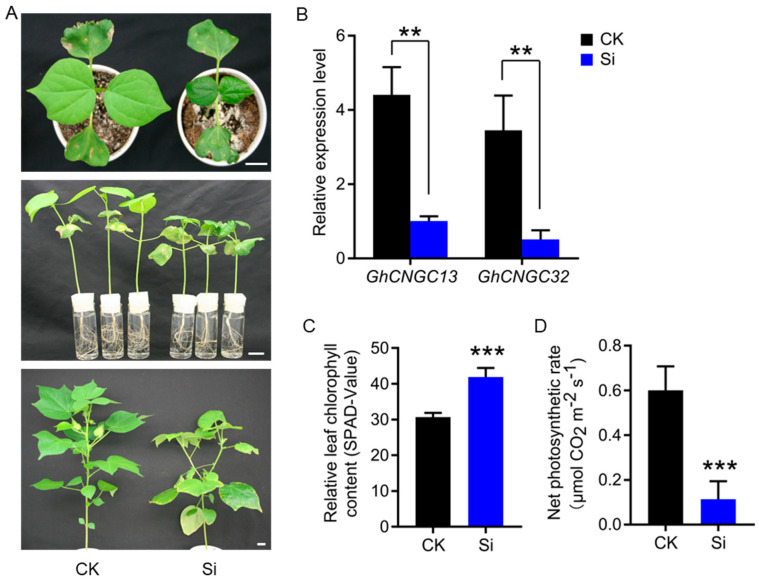
Silencing *GhCNGC13* and *32* suppressed cotton growth and development. (**A**) Phenotypic symptoms of *GhCNGC13*- and *32*-silenced (Si) plants 14 d (upper panel), 17 d (middle panel), or 62 d (bottom panel) after VIGS treatment compared to mock control (CK) plants. Scale bar = 2 cm. (**B**) Relative expression levels of *GhCNGC13* and *32*. Leaf samples 21 d after VIGS were detected by quantitative RT-PCR using *GhUBQ7* as an internal control. (**C**) Relative leaf chlorophyll contents. (**D**) Leaf net photosynthetic rates. Data are means ± SD (n = 3 for (**B**); 6 for (**C**); 5 for (**D**)), Student’s *t*-test: ** *p* < 0.01, *** *p* < 0.001.

**Figure 2:**
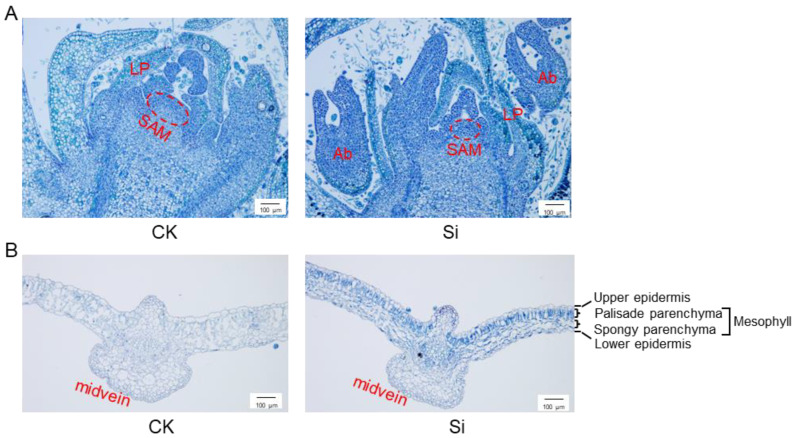
Silencing *GhCNGC13* and *32* caused anatomical changes in cotton shoot tips and leaves. (**A**) Longitudinal sections of the main shoot tip, showing the arrested growth of the SAM (dashed oval) and concomitant growth of axillary buds in *GhCNGC13*- and *32*-silenced (Si) versus mock control (CK) plants 21 d after VIGS treatment. SAM, shoot apical meristem; Ab, axillary bud; LP, leaf primordium. (**B**) Transverse sections of the 2nd leaf of Si versus CK plants 21 d after VIGS. Sections were stained with toluidine blue and observed under a light microscope. Scale bar = 100 µm.

We have previously identified a total of 40 genes encoding CNGCs in the genome of Upland cotton [27]. Of them, *GhCNGC13* and *32* are the homologous gene pair from the A- and D-subgenome, encoding almost identical amino acid sequences (97.8% identity and 99.0% similarity) that represent the closest homologs to AtCNGC2; moreover, they have very similar expression patterns, typically being highly expressed in all tissues [27]. To obtain insights into the biological functions of *GhCNGC13* and *32*, we performed a virus-induced gene silencing (VIGS) assay to simultaneously knock down both genes in planta. In comparison with mock control plants, *GhCNGC13*- and *32*-silenced plants exhibited leaf initiation and expansion arrest, plant height reduction, root system suppression, and main shoot apical growth abortion (Figure 1A). Transcript levels of *GhCNGC13* and *32* were reduced by 77% and 85% in the leaves of silenced plants, respectively (Figure 1B). It was noted that leaves in the silenced plants became darker in color (Figure 1A, upper panel), which agreed with increased chlorophyll contents (Figure 1C); however, the net photosynthetic rate was significantly reduced (Figure 1D), suggesting that *GhCNGC13* and *32* are functionally linked with photosynthesis. The main stem of cotton has an erect and indeterminate monopodial growth habit, as seen in control plants (Figure 1A, bottom-left panel), but *GhCNGC13*- and *32*-silenced plants showed branches growing due to shoot tip growth abortion (Figure 1A, bottom right panel). Further examination of the microscopic anatomy confirmed that the main shoot apical meristem (SAM) of silenced plants was reduced in size, whereas axillary buds appeared larger (Figure 2A). These results suggest that *GhCNGC13* and *32* are required for SAM development, a vital control center for plant growth and development. In *Arabidopsis*, *AtCNGC2* loss-of-function mutant *dnd1* plants are dwarfed in stature and have rosette leaves of reduced sizes [35]. *dnd1* plants also show earlier natural senescence (i.e., the tips of leaves begin turning yellow) [36] and a significantly delayed flowering phenotype [37]. Interestingly, previous studies have suggested that AtCNGC2-mediated Ca^2+^ signaling is linked with the CLAVATA3/CLAVATA1 (CLV3/CLV1) signaling cascade that influences *WUSCHEL* (*WUS*) and *FANTASTIC FOUR 2* (*FAF2*) expression, stem cell fate, and ultimately the size of the SAM [38]. Taken together, *GhCNGC13* and *32* have similar functions to *AtCNGC2* in the control of plant growth and development. 

*GhCNGC13*- and *32*-silenced plants produced leaves with a reduced leaf thickness, smaller and more densely packed mesophyll cells, reduced intercellular airspaces within the palisade and spongy parenchyma, obviously due to cell growth arrest; in addition, the mesophyll tissues were stained darker (Figure 2B), which may indicate alterations in cell wall structure properties and/or cellular acidic components (e.g., nucleic acids, carboxylates) [39,40]. Thus, silencing *GhCNGC13* and *32* impaired the leaf anatomic structures underlying photosynthetic performance and the related physiological processes, which agreed with the reduced leaf net photosynthetic rate (Figure 1D). It has been previously documented that antisense suppression of *AtCNGC10* caused a reduction in leaf surface area and thickness and palisade parenchyma cell length, but increased starch accumulation in the chloroplasts [41]. Nevertheless, the potential link between CNGCs and photosynthesis is rarely mentioned in the literature.

### 2.2. GhCNGC13 and 32 Are Functionally Linked with Plant Immunity

**Figure 3:**
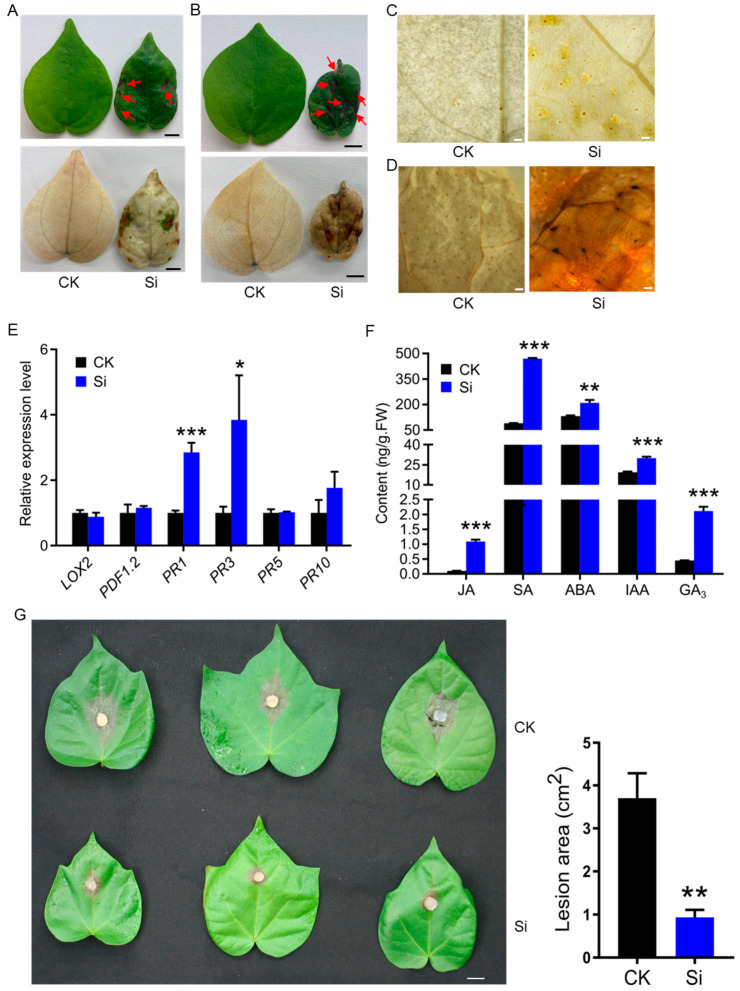
Silencing *GhCNGC13* and *32* triggered plant autoimmunity. (**A**) Leaf spontaneous necrotic spotting (indicated by arrows; upper panel) and DAB (3,3′-diaminobenzidine) staining for detection of hydrogen peroxide accumulation (brown spots; lower panel). The first leaves were sampled from *GhCNGC13*- and *32*-silenced (Si) or mock control (CK) plants 21 d after VIGS treatment. Scale bar = 0.5 cm. (**B**) Trypan blue staining for detection of cell death (blue color; lower panel). See (**A**) for a sampling explanation. Scale bar = 0.5 cm. (**C**,**D**) Higher magnification of a portion of the leaf blade stained by DAB in (**C**) or trypan blue in (**D**). Scale bar = 1 mm. (**E**) Relative transcript levels of defense-related marker genes. Quantitative RT-PCR analyses were performed using *GhUBQ7* as an internal control. (**F**) Phytohormone contents in the shoot samples (excluding cotyledons) of plants 14 d after VIGS. (**G**) Evaluation of resistance against *Botrytis cinerea*. Left panel, representative leaves three days after inoculation; right panel, measurement of lesion size. Scale bar = 1 cm. JA, jasmonate; SA, salicylic acid: ABA, abscisic acid; IAA, indole-3-acetic acid; GA_3_, gibberellin. Data are means ± SD for three biological replicates in (**E**) and (**G**) or three technical repeats in (**F**), Student’s *t*-test: * *p* < 0.05, ** *p* < 0.01, *** *p* < 0.001.

AtCNGC2 is known to be involved in signal transduction pathways related to hypersensitive responses (HRs) and immunity [42,43]. *AtCNGC2* loss-of-function mutants (*dnd1*) exhibit loss-of-HR phenotypes; however, leaf lesions are frequently observed in plants [35], a phenomenon known as autoimmunity [44]. Similarly, we found that *GhCNGC13*- and *32*-silenced plants displayed necrotic spotting or lesions (Figure 3A,B; upper panels). It has been demonstrated that pathogen attack can activate AtCNGC2 to cause cytosolic Ca^2+^ elevation, which is linked to NO (nitrogen oxidize) and reactive oxygen species (ROS) generation, leading to various downstream responses [42]. Consistently, we confirmed H_2_O_2_ accumulation via DAB staining in the leaves of *GhCNGC13*- and *32*-silenced plants (Figure 3A,C), as well as obvious cell death via trypan blue staining (Figure 3B,D). 

The *dnd1* plants retain characteristic defense responses such as induction of pathogenesis-related (PR) gene expression and strong restriction of pathogen growth [45]; moreover, *dnd1* mutation causes constitutive elevations in salicylic acid (SA) compounds and systemic acquired resistance (SAR) [35,45]. We found that *PR1* and *PR3* were significantly upregulated in *GhCNGC13*- and *32*-silenced plants, while no significant expression changes of *PR5*, *PR10*, *PDF1.2*, and *LOX2* were detected (Figure 3E). PR proteins are the key components of the plant innate immune system, especially SAR, and they are widely used as diagnostic molecular markers of defense signaling pathways [46]. Increased expression of *PR1* may indicate activation of the SA signaling pathway, whereas elevated expression of *PR3* indicates activation of the jasmonic acid (JA) pathway [46]. We confirmed that both SA and JA contents significantly increased in *GhCNGC13*- and *32*-silenced plants; however, abscisic acid (ABA), auxin (indole-3-acetic acid; IAA), and gibberellic acid (GA_3_) contents were also elevated (Figure 3F). Very recently, AtCNGC2 has been found to modulate auxin homeostasis and signaling, and the loss-of-function mutations exhibit higher levels of IAA [21], in addition to elevated SA basal levels [35,45]. Phytohormones function as a network by auto-regulating themselves and cross-regulating each other via acting as negative feedback regulators in plants [47,48,49]. It has been noted that phytohormone contents display dynamic changes in plants, and thus can be only measured to represent endpoint results [50]. Generally, IAA and GA_3_ play prominent roles in the control of plant growth and development, whereas ABA functions mainly in regulating stress responses and adaptation [51]. While SA and JA are well recognized as major defense hormones, many other hormones including ABA, auxin, and gibberellins have been reported to modulate the plant immune signaling network as well [52]. 

Autoimmune activation usually confers an enhanced resistance to pathogens in plants [44]. Thus, we examined the defense response of *GhCNGC13*- and *32*-silenced plants against pathogen infection. An air-borne fungal pathogen *Botrytis cinerea*, the cause of gray mold disease, was used to inoculate the leaves. Consequently, *GhCNGC13*- and *32*-silenced plants produced much smaller necrotic areas from the infection compared to the non-silenced control plants (Figure 3G). This result confirmed an enhanced resistance to *B. cinerea* in the silenced plants, most likely benefiting from the autoimmunity. Taking all the above results together, we concluded that GhCNGC13 and 32 are tightly linked with the plant immune system, which may involve actions of both stress and growth hormones. Intriguingly, recent advances support that cAMP and cGMP, the presumed activators of CNGC channel activities, interact with various phytohormones in regulating plant growth, development, and stress responses [53,54,55]. Hence, GhCNGC13 and 32 could be important nodes in generating signaling crosstalk between cyclic nucleotides (cAMP and cGMP), Ca^2+^, and phytohormones, which might reflect a complex and delicate regulatory system of plant growth and adaptation or immunity. 

### 2.3. Silencing GhCNGC13 and 32 Compromises Cotton Verticillium Wilt Resistance

**Figure 4:**
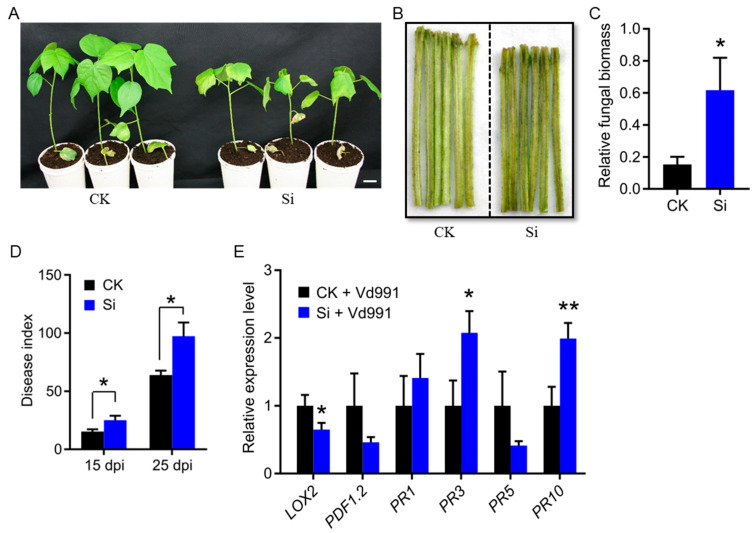
Silencing *GhCNGC13* and *32* compromised cotton *Verticillium* wilt resistance. (**A**) Phenotypic symptoms of *GhCNGC13*- and *32*-silenced (Si) versus mock control (CK) plants after inoculation of *V. dahliae* strain Vd991 14 d after VIGS treatment. Photographs were taken 14 days post-inoculation (dpi). (**B**) Vascular wilt symptoms 20 dpi. Scale bar = 2 cm. (**C**) Relative fungal biomass in the stem tissues 14 dpi. (**D**) Disease index. The results were averaged from two independent experiments, one-tailed Student’s *t*-test: * *p* < 0.05. (**E**) Relative transcript levels of defense-related marker genes. Quantitative RT-PCR was performed using *GhUBQ7* as an internal control. Data in (**C**,**E**) are means ± SD for three biological replicates, two-tailed Student’s *t*-test: * *p* < 0.05, ** *p* < 0.01.

*Verticillium* wilt is among the most yield-limiting diseases in cotton, and is caused by the soil-borne fungus *Verticillium dahlia*. Typically, infected plants display obvious symptoms including vascular discoloration, leaf chlorosis, curling, or necrosis, followed by defoliation and eventually wilting and plant death as the disease progresses [56]. To assess the practical promise of autoimmunity in improving cotton resistance against *Verticillium* wilt, we inoculated *GhCNGC13*- and *32*-silenced and mock control plants with *V. dahliae* stain Vd991 via the root-dipping method. Surprisingly, *GhCNGC13*- and *32*-silenced plants showed an earlier onset of leaf chlorosis and wilting symptoms compared to mock control plants (Figure 4A). Further analyses confirmed that *GhCNGC13*- and *32*-silenced plants developed more severe symptoms of stem vascular discoloration (Figure 4B), a higher fungal biomass in the stem tissues (Figure 4C), and a higher disease index (Figure 4D). Together, all these data indicate that silencing *GhCNGC13* and *32* caused compromised *Verticillium* wilt resistance in Upland cotton. Thus, autoimmune activation in the silenced plants (Figure 3) was not enough to resist *Verticillium* wilt. We detected the same set of defense-related marker genes as described above (Figure 3E), showing that *PR3* and *PR10* were significantly upregulated, whereas *LIPOXYGENASE2 (LOX2)* was significantly downregulated in the silenced plants upon Vd991 inoculation (Figure 4E). *LOX2* encodes an important enzyme in the initial steps of JA biosynthesis [57]. It was reported that *LOX2* is strongly induced by *V. dahliae* inoculation or methyl jasmonate (MeJA) treatment and that *LOX2* knock-down enhances cotton susceptibility to *V. dahliae* Vd991 [58]. Therefore, the compromised *Verticillium* wilt resistance of *GhCNGC13*- and *32*-silenced plants was at least in part due to *LOX2* downregulation. This supports the notion that *GhCNGC13* and *32* may act through *LOX2* to play a critical role in positively regulating the *Verticillium* wilt resistance of cotton. 

### 2.4. Identification and Functional Enrichment Analyses of DEGs Associated with GhCNGC13 and 32 Silencing

**Figure 5:**
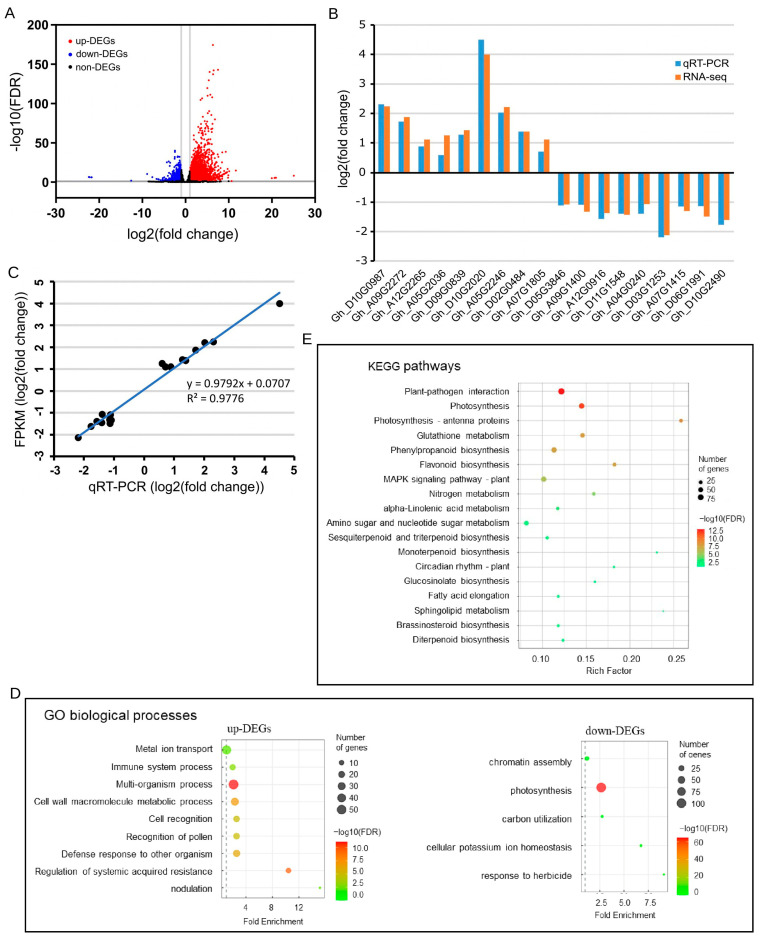
Identification and functional enrichment analyses of DEGs associated with *GhCNGC13* and *32* silencing. (**A**) Identification of differentially expressed genes (DEGs; Supplementary Table S2) by RNA-seq analysis of *GhCNGC13*- and *32*-silenced versus mock control plants 17 d after VIGS treatment. (**B**) Verification of RNA-seq data by qRT-PCR. (**C**) Correlation between the results of qRT-PCR and RNA-seq as shown in (**B**). (**D**) Functional enrichment of DEGs with GO biological processes at the level of FDR (*q*-value) < 0.05. (**E**) Functional enrichment of DEGs with KEGG pathways at the level of FDR < 0.05. GO, Gene Ontology; KEGG, Kyoto Encyclopedia of Genes and Genomes.

To comprehensively understand the molecular basis underlying the biological functions of GhCNGC13 and 32, we performed an RNA-seq analysis using whole-plant tissue samples (excluding the cotyledons) of *GhCNGC13*- and *32*-silenced versus mock control plants. The assembled reads resulted in 73,344 genes (including 14,618 novel genes). Based on the estimated expression levels of FPKM (fragments per kilobase of exon per million mapped fragments), differentially expressed genes (DEGs) were determined at the threshold of |log2(FoldChange)| > 1 and FDR-adjusted *p*-value (*p*_adj_) of < 0.05. Consequently, a total of 4835 DEGs (including 866 novel genes) were identified, accounting for 6.6% among all the detected genes in the genome, with 3546 of them upregulated and 1289 downregulated (Figure 5A; Supplementary Table S2). This result supported that *GhCNGC13* and *32* have a widespread impact on Upland cotton. We validated the reliability of RNA-seq data by qRT-PCR detection of 18 randomly selected up- and downregulated DEGs using gene-specific primers (Supplementary Table S1), showing a highly significant correlation (R^2^ = 0.9776, *p* < 0.001) between the results of RNA-seq and qRT-PCR (Figure 5B,C). 

The above DEGs were subjected to GO (Gene Ontology) enrichment analysis, indicating their functional involvement in a wide range of biological processes (Supplementary Table S3). After removing redundant terms among the significantly enriched biological processes using REVIGO [59], as shown in Figure 5D, the results showed that the upregulated DEGs mainly function in plant immunity (regulation of system-acquired resistance; defense response to other organisms; immune system process), interactions between cells or organisms (multi-organism process; nodulation; cell recognition; recognition of pollen), cell wall macromolecule metabolic processes, and metal ion transport. In contrast, the downregulated DEGs are primarily implicated in photosynthesis, carbon utilization, cellular potassium ion homeostasis, and chromatin assembly. A KEGG (Kyoto Encyclopedia of Genes and Genomes) pathway enrichment analysis indicated that the plant–pathogen interaction pathway and the photosynthesis pathway were the two most significantly enriched pathways at the threshold of FDR < 0.05 (Figure 5E; Supplementary Table S4). These DEGs provided a molecular basis not only for the essential role of *GhCNGC13* and *32* in plant growth, closely related to photosynthesis (Figure 1 and Figure 2), but also for their important role in plant immunity (Figure 3). Given the fact that plant autoimmunity was induced simultaneously with growth inhibition in *GhCNGC13*- and *32*-silenced plants, we concluded that *GhCNGC13* and *32* are key players in balancing plant growth and immunity, thus acting in the so-called “growth–defense tradeoff” phenomenon widely observed in crops [60,61]. 

### 2.5. Transcription Factors Associated with GhCNGC13 and 32

**Figure 6:**
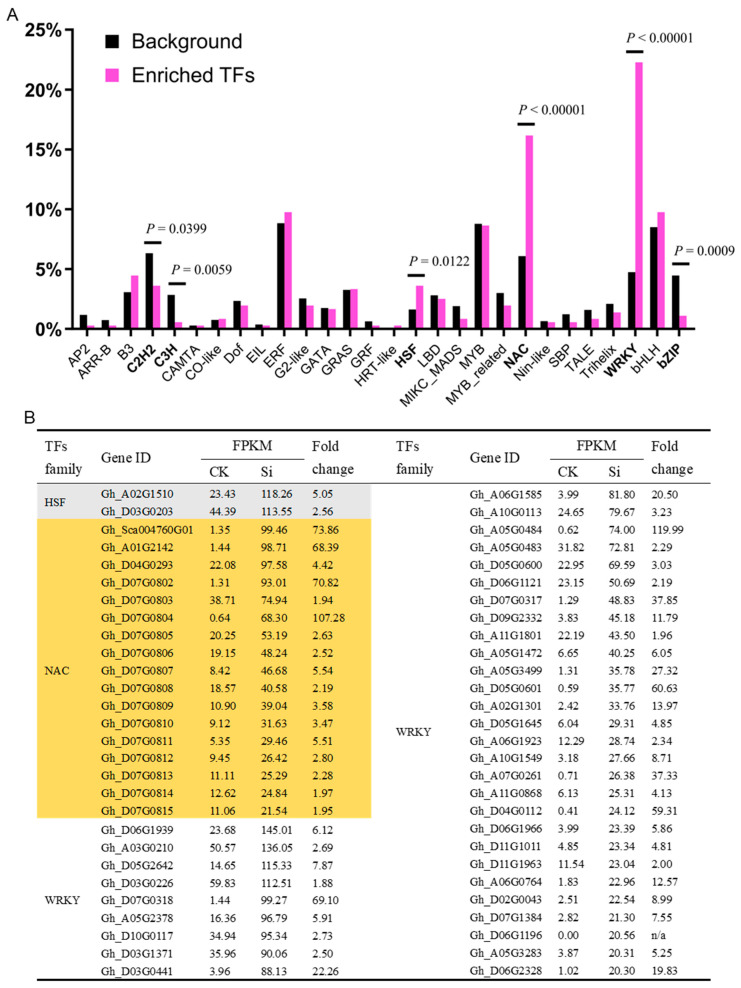
Transcription factor (TF) families of DEGs associated with *GhCNGC13* and *32* silencing. (**A**) Over- and under-representation analyses of TF families in the DEGs. Fisher’s exact test was performed to determine *p*-values for the significance of difference. (**B**) List of representative members of the over-represented TFs families. Only those genes with an average FPKM of > 20 in either the sample of *GhCNGC13-* and *32*-silenced (Si) or mock control (CK) plants are listed. See the legend in Figure 5 for an explanation of RNA-seq analysis. DEGs, differentially expressed genes; FPKM, fragments per kilobase of exon model per million mapped fragments.

A large number of transcription factors (TFs), including different structural and functional families such as CAMTA, MYB, WRKY, bZIP, and NAC, are regulated by Ca^2+^ at different levels, from direct binding of Ca^2+^, CaM, or other Ca^2+^ sensors to modification by Ca^2+^-regulated protein kinases and phosphatases [62]. TFs are important components of plant signaling pathways that define plant responses to biotic and abiotic stimuli, as well as developmental cues [63], and they may function at the intersection of plant growth and immunity [60]. Therefore, we performed over- and under-representation analyses of TF families in the above-identified DEGs. The results showed that HSF (heat shock factor), NAC (NAM, ATAF, and CUC), and WRKY families were significantly over-represented, while C2H2, C3H, and bZIP families were significantly under-represented (Figure 5A). A total of 13, 58, and 80 DEGs belong to the HSF, NAC, and WRKY families, respectively. They account for 15.9%, 19.0%, and 33.6% of the total HSF (82), NAC (306), and WRKY (238) family members deposited in the plant TF database [64]. The Upland cotton genome contains more than 80 HSFs [65], 283 NAC TFs [66], and 239 WRKY genes [67]. HSFs play crucial roles not only in biotic and abiotic stresses but also in plant development [68,69]. It has been reported that CNGCs play a key role in initiating the heat-stress response by regulating HSFs [70,71]. NAC TFs are a plant-specific family involved in multiple biological processes, including biotic and abiotic stress responses, growth, and development [72]. The association between CNGCs and NAC TFs has not been documented in the literature until now. WRKY TFs are among the largest families of transcriptional regulators found exclusively in plants, and they are well known for regulating many aspects of plant growth and development (e.g., stem elongation, root development, reproductive development, leaf senescence, cellular homeostasis, embryogenesis, seed and trichome development, nutrient deprivation and utilization), hormonal signaling, metabolic pathways, and diverse biotic and abiotic stress responses [63,73,74]. The latest discoveries have illustrated the interaction of WRKY proteins with other TFs to form an integral part of signaling webs that regulate several seemingly disparate processes and defense-related genes, thus establishing their significant contributions to the plant immune response [63]. WRKY TFs have long been suggested as being part of the plant–pathogen interaction network and may act downstream of CNGCs [75]. Intriguingly, different WRKY transcription factor binding sites have been identified in almost all the promoter sequences of CNGCs in different plants, including cotton [27,76,77], indicating that CNGCs may also be regulated by WRKY TFs. Here, we listed a set of representative DEGs in the HSF, NAC, and WRKY families (Figure 6B) which may provide key targets for facilitating further dissection of *GhCNGC13-* and *32*-mediated signaling pathways. 

### 2.6. GhCNGC13 and 32 Are Crucial Modulators of the Plant–Pathogen Interaction Pathway

**Figure 7:**
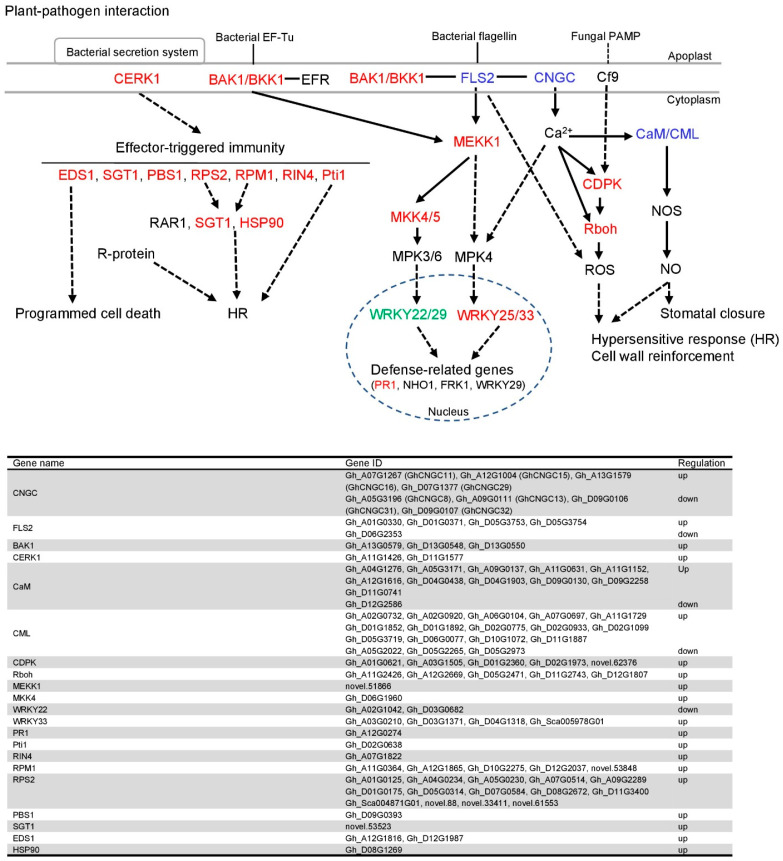
The plant–pathogen interaction pathway annotated with DEGs in association with *GhCNGC13* and *32* silencing. RNA-seq analysis was performed to determine differentially expressed genes (DEGs; Supplementary Table S2) between *GhCNGC13*- and *32*-silenced and mock control plants. The pathway is schematically depicted following a KEGG pathway map (https://www.genome.jp/entry/map04626 (accessed on 4 May 2023)), showing homologous genes of the upregulated DEGs in red, the downregulated in green, and the up/downregulated in blue. Information on the annotated DEGs is listed in the bottom panel.

Calcium signaling plays a fundamental role in plant immune responses [78]. AtCNGC2 has been demonstrated to be a key player in mediating Ca^2+^ signaling in the modulation of plant immunity [42]. Consistently, as shown in Figure 7, most genes in the KEGG plant–pathogen interaction pathway were annotated by DEGs in association with *GhCNGC13* and *32* silencing; moreover, most of them were upregulated. These DEGs are involved in (1) the perception of pathogens by cell surface pattern-recognition receptors (PRRs) or the so-called PAMP-triggered immunity (PTI), including *FLS2* (*FLAGELLIN-SENSITIVE 2*), *BAK1* (*brassinosteroid insensitive 1-associated receptor kinase 1*)*/BKK1* (*BAK1-LIKE 1*), and *CERK1* (*chitin elicitor receptor kinase 1*); (2) the secondary response or the so-called effector-triggered immunity (ETI), including *EDS1* (*ENHANCED DISEASE SUSCEPTIBILITY 1*), *SGT1* (*suppressor of the G2 allele of Skp1*), *PBS1* (*AVRPPHB SUSCEPTIBLE 1*), *RPS2* (*RESISTANT TO P. SYRINGAE 2*), *RPM1* (*RESISTANCE TO P. SYRINGAE PV MACULICOLA 1*), *RIN4* (*RPM1 INTERACTING PROTEIN 4*), and *Pti1* (*Pto-interacting protein 1*); (3) downstream activation, including *MEKK1* (*mitogen-activated protein kinase kinase 1*), *MKK4/5* (*mitogen-activated protein kinase kinase 4/5*), *WRKY25/33* (*WRKY transcription factor 25/33*), *WRKY22/29*, *PR1*, *CDPK* (*calcium-dependent protein kinase*), *Rboh* (*respiratory burst oxidase homolog*), *HSP90* (*heat shock protein 90*), *CaM* (*calmodulin*), and *CML* (*calmodulin-like*). This result may provide a molecular basis for the autoimmunity observed in *GhCNGC13*- and *32*-silenced plants (Figure 3). 

Very interestingly, while *GhCNGC13* and *32* were among the DEGs, many other members of the *GhCNGC* family were also among the DEGs, including upregulation of *GhCNGC11*, *15*, *16*, and *29* and downregulation of *GhCNGC8* and *31*. This result indicated that *GhCNGC13* and *32* interplay with other members of the *GhCNGC* family. It has been demonstrated that plant CNGCs form hetero-tetrameric complexes in which the combination of subunits may confer unique functional characteristics to the channel compared to homo-tetrameric complexes [3,29,79,80]. The formation of channel complexes depends on the co-localization and relative abundance of CNGC isoforms [3]. Previous reports have shown in planta interactions between AtCNGC2 and AtCNGC4 [28,79], between AtCNGC8 or AtCNGC7 and AtCNGC18 [29], and between AtCNGC19 and AtCNGC20 [80], as well as among AtCNGC6, AtCNGC9, and AtCNGC14 [17]. Additionally, *AtCNGC11* and *AtCNGC12* may act synergistically in multiple Ca^2+^-dependent physiological responses [81]. Therefore, we postulated that the orchestrated regulation among the CNGCs family members represents a universal and important regulatory mechanism in plants.

### 2.7. GhCNGC13 and 32 Are Important Players in Regulating the Photosynthesis Pathway

**Figure 8:**
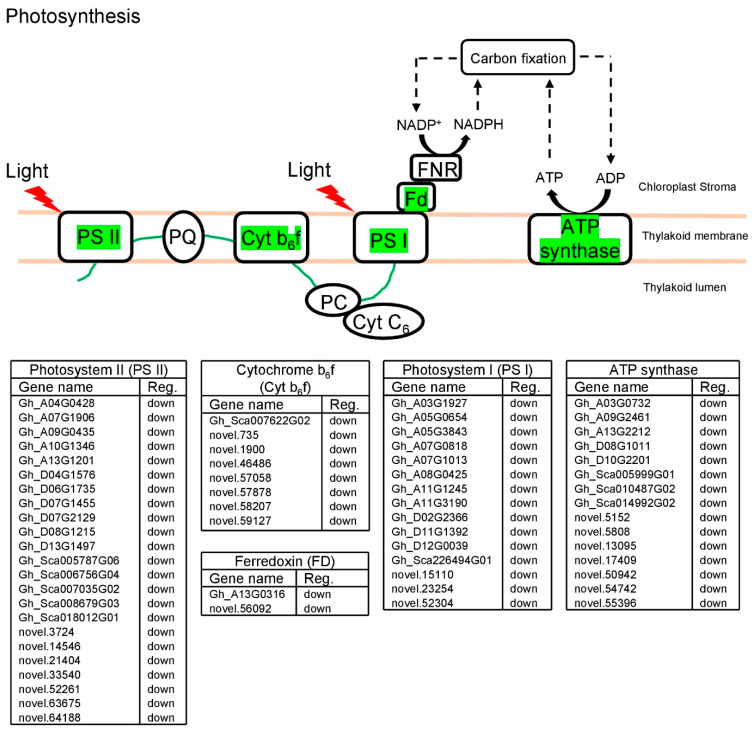
Photosynthesis pathway annotated with DEGs in association with *GhCNGC13* and *32* silencing. RNA-seq analysis was performed to determine differentially expressed genes (DEGs; Supplementary Table S2) between *GhCNGC13*- and *32*-silenced and mock control plants. The pathway is schematically depicted following the KEGG pathway map (https://www.genome.jp/entry/map00195 (accessed on 4 May 2023)), highlighting homologous genes of the DEGs in green, which were all downregulated. Information on the annotated DEGs is listed in the bottom panels. PQ, plastoquinone; PC, plastocyanin; FNR, ferredoxin-NADP^+^ reductase; NADP^+^/NADPH, nicotinamide adenine dinucleotide phosphate (oxidized/reduced form); ATP, adenosine triphosphate; ADP, adenosine diphosphate; Cyt C_6_, cytochrome C_6_.

Most members of the CNGC family in a plant species, including cotton, are predicted to be localized in the plasma membrane, but a few other members may reside in the chloroplasts or other intracellular organelles [27]. For example, AtCNGC13 has been identified in the purified stroma thylakoids from leaves [82]. Chloroplasts are important sites for Ca^2+^ signaling [83]. Moreover, calcium signals are constantly generated as a downstream effect of the circadian clock that modulates many biological processes including photosynthesis, carbon metabolism, growth, and water use [62]. As shown in Figure 8, many DEGs (including 38 known genes) were annotated in the photosynthetic light reactions, with their functions related to photosystem II (PS II), cytochrome b_6_f complex (Cyt b_6_f), photosystem I (PS I), ATP synthase, and ferredoxin (FD), which were all downregulated. Thus, silencing *GhCNGC13* and *32* suppressed photosynthetic light reactions and the leaf net photosynthetic rate (Figure 1D), although leaf chlorophyll contents were increased (Figure 1C). These results supported that *GhCNGC13* and *32* are crucial players in maintaining photosynthesis, which may represent an important mechanism for establishing the mutual connection between plant growth and photosynthesis. 

GhCNGC13 and 32, like AtCNGC2, are localized in the plasma membrane (our unpublished data), which may rule out direct impacts on photosynthesis. However, AtCNGC2 is well known to mediate Ca^2+^ flux and signaling [24,42,79,84,85], and it has also been demonstrated to interplay with a variety of signaling events, including cyclic nucleotides (cAMP and cGMP) [86], phytohormones (IAA, JA, and SA) [21,24,35], NO [36], CLV3/CLV1 [38], and light [87]. Thus, GhCNGC13 and 32 may constitute the key components of a complex signaling network and have significant impacts on plant growth and photosynthesis. cAMP, a prime activator of the CNGC family, has been recently recognized as an essential signaling molecule in plants where cAMP-dependent processes include responses to hormones and environmental stimuli [88]. Silencing *GhCNGC13* and *32* caused altered expression of several other family members, including *GhCNGC11*, *15*, and *19* (Figure 7), all of which encode the closest homologs of AtCNGC13 [27]. AtCNGC13 has been found in the stroma thylakoid of chloroplasts [82]. It will be interesting to investigate whether *GhCNGC11*, *15*, and *19* also reside in the chloroplasts. Recent reports have highlighted the central role of chloroplasts in integrating various environmental signals and regulating plant immunity by transmitting signals to the nucleus and other cell compartments through retrograde signaling pathways [83,89,90,91]. Chloroplasts are the site of the synthesis of secondary metabolites and defense compounds, as well as phytohormones such as JA and SA [89,91]. In addition, chloroplasts are major generators of ROS and NO, which are essential defense molecules [83]. Photosynthesis depends on the activity of chloroplast ion channels, and it has emerged that chloroplasts may play an important role in programmed cell death (PCD) in plant leaves [92,93]. Furthermore, alteration of the chloroplast ion homeostasis may lead to organelle dysfunction, which in turn significantly affects the energy metabolism of the whole organism [94]. Overall, we postulated that *GhCNGC13* and *32* provide the key molecular link between plant growth and immunity. 

## 3. Materials and Methods

### 3.1. Plant Materials and Growth Conditions

All plants used in this study were Upland cotton cultivar “0–153” as described previously [27]. The seeds were grown in potting soil (Pindstrup Mosebrug A/S, Ryomgård, Denmark) or cultivated hydroponically using Hoagland’s nutrient solution in a growth room at 25 °C with a 16 h light/8 h dark photoperiod. 

### 3.2. VIGS Assay

Virus-induced gene silencing (VIGS) is an RNA interference-based technique that has been widely used in cotton and many other plant species to transiently knock down target gene expression by utilizing modified plant viral genomes [95]. Tobacco rattle virus (TRV) invades a wide range of hosts and can spread vigorously throughout the entire plant [96]. Here, agrobacterium-mediated VIGS was performed by utilizing a TRV-based binary vector system as follows. A partial cDNA fragment conserved between *GhCNGC13* and *GhCNGC32* was selected for target specificity within the genome and then cloned into the binary vector pTRV2. The primers used for cloning are listed in Supplementary Table S1. The resulting plasmid pTRV2-*GhCNGC13* and *32* were introduced into *Agrobacterium tumefaciens* GV3101. Cotton plants were prepared to grow at the stage of two fully expanded cotyledons about two weeks old. A mixture of an agrobacterial culture suspension of pTRV1 and pTRV2-*GhCNGC13* and *32* in a 1:1 ratio was infiltrated into cotyledons from the underside using a needleless syringe. Meanwhile, agrobacterial infiltration of pTRV1 combined with the empty pTRV2 was used as a negative (mock) control, and pTRV1 combined with pTRV2-*PDS* was used as a positive control. *Phytoene desaturase* (*PDS*) silencing resulted in an albino phenotype on new leaves, generally seen about 2 weeks post agroinfiltration, providing a visual marker for silencing efficiency. Once the silencing of *PDS* was consistent, corresponding plants from pTRV2 and pTRV2-*GhCNGC13* and *32* plants were used for experiments. A detailed description of the above VIGS procedure is in our previous publication [27]. All the experiments were conducted at least three times independently, and similar results were obtained.

### 3.3. Leaf Chlorophyll Measurement

Leaf chlorophyll contents were determined by measuring SPAD values using a SPAD-502Plus meter (Konica Minolta, Tokyo, Japan), which can provide accurate, quick, and non-destructive in situ measurements of relative leaf chlorophyll contents [97]. Six plants per treatment were measured with the first expanded leaf, and an average value was obtained for each leaf in three independent measurements. Two independent experiments with similar results were conducted. 

### 3.4. Leaf Net Photosynthetic Rate Measurement

Leaf net photosynthetic rate (*P*_n_) measurements were performed using an LCpro-SD (ADC Bioscientific, Hoddesdon, UK) portable photosynthesis system analyzer [98]. Five plants per treatment were measured with the first expanded leaf, and duplicate measurements were performed for each leaf. Two independent experiments were performed with similar results.

### 3.5. Quantitative RT-PCR

Total RNA was isolated using an EASYspin Plus Plant RNA Kit (Aidlab, Beijing, China). Reverse transcription (RT) was performed to synthesize cDNA using a HiScript III RT SuperMix for qPCR Kit (Vazyme, Nanjing, China). Quantitative PCR analysis was carried out using ChamQ Universal SYBR qPCR Master Mix (Vazyme) in a LightCycler^®^ 480II PCR system (Roche, Basel, Switzerland). The cycling conditions were 30 s at 95 °C, 40 cycles of 10 s at 95 °C, and 30 s at 60 °C. Three replicates were conducted for each reaction. All the primers are listed in Supplementary Table S1. Amplification products were verified via a melting curve analysis, followed by electrophoresis on 1.8% agarose gel. Relative expression levels were calculated using the 2^−ΔΔCq^ method, normalized to *GhUBQ7* (GenBank: DQ116441). 

### 3.6. Histology

The experiments were performed at Wuhan Servicebio Technology (Wuhan, China) following a standard laboratory procedure [99]. The main shoot apex or leaf samples were dissected and then fixed in a formalin–acetic acid–alcohol (FAA) solution overnight. Tissues were dehydrated and cleared using a series of ethanol and/or xylene, and finally transferred to refined paraffin wax. Serial sections were cut using a microtome, mounted on slides, deparaffinized, and stained with toluidine blue. Images were captured using a compound light microscope (Zeiss, Oberkochen, Germany).

### 3.7. DAB Staining

By following a previously described protocol [100], leaves were collected and submerged in a fresh preparation of 3,3′-diaminobenzidine (DAB) staining solution (1 mg/mL, pH 3.0). After applying a gentle vacuum for 5 min, the leaves were stained by incubation at room temperature with shaking (~80 rpm) in the dark for 4~5 h. Subsequently, the DAB staining solution was replaced with a bleaching solution (ethanol:acetic acid:glycerol = 3:1:1) and placed in a boiling water bath for about 15 min until the leaves were free of chlorophyll. Finally, after replacement with fresh bleaching solution, it was allowed to stand for 30 min. The appearance of brown precipitates in the leaves indicated the presence of a H_2_O_2_ burst. The experiment was conducted twice with similar results.

### 3.8. Trypan Blue Staining

As described in a previous report [101], leaves were immersed in a fresh preparation of trypan blue staining solution (25% phenol (TE buffer equilibrated, pH 7.5~8.0), 25% glycerol, 25% lactic acid (85% *w*:*w*), trypan blue 10 mg/mL) and incubated for 30~60 min. Then, the staining solution was replaced with 98~100% ethanol and incubated overnight, followed by repeated replacement with fresh ethanol until green tissue became completely colorless. The leaves were finally kept in a 60% glycerol solution for microscopy purposes. A blue color indicated dead tissue. Similar results were obtained in two independent experiments.

### 3.9. Quantitation of Phytohormones

Phytohormones were measured as we described in detail previously [55]. Plant tissue samples were extracted with methanol. After purification, the samples were analyzed in triplicate using an AB SCIEX Q-TRAP^®^ 6500 LC-MS/MS system (Applied Biosystems, Waltham, MA, USA). Phytohormone contents were calculated according to standard curves that were prepared using a serial dilution of analytical phytohormone standards from Sigma-Aldrich (St. Louis, MO, USA).

### 3.10. Determination of Botrytis cinerea Resistance

The experiment was performed following a previous publication [102]. The *Botrytis cinerea* strain was taken from storage at 4 °C and transferred onto a potato-dextrose agar (PDA) medium for four days, and then high-activity hyphae were transferred into fresh PDA medium for another seven days to enable spores to form. *B. cinerea* was prepared with identical virulence in concentric circles using a punch (5 mm in diameter) and used to inoculate the third leaf collected from cotton seedlings eight weeks after VIGS treatment. The infected leaves were photographed after three days, and the size of necrotic lesions was measured in ImageJ software v1.53 (http://rsbweb.nih.gov/ij/ (accessed on 5 October 2023)). The experiment was conducted twice with similar results. 

### 3.11. Analysis of Verticillium wilt Resistance

Plants were subjected to a Verticillium wilt resistance test about 3 weeks after VIGS treatment. The highly virulent defoliating strain Vd991 of *Verticillium dahlia*, a soil-borne pathogenic fungus causing *Verticillium* wilt in cotton, was prepared for inoculation as we described previously [27]. Each plant grown in a 9-ounce paper cup was inoculated with 30 mL of conidial suspension (1 × 10^7^ conidia/mL) by the root infection method. The vascular wilt symptom was investigated under a microscope by cutting the main stems after inoculation. For in planta fungal biomass quantification, genomic DNA was isolated from the stems of inoculated plants, and then quantitative PCR was performed using primers (Supplementary Table S1) specific to the internal transcribed spacer region (ITS) of the 5.8S ribosomal RNA gene in *V. dahliae* and *GhUBQ7* in *G. hirsutum* for sample equilibration. The ratio of fungus to plant DNA was calculated using the 2^−ΔΔCq^ method. The plant disease index (DI), which reflects the disease infection status of a population, was determined according to a previous report [103]: the severity of disease symptoms on each cotton seedling was rated into five levels, wherein 0 = no visible wilting or yellowing symptoms, 1 = one or two leaves wilted or dropped off, 2 = three or four leaves wilted or dropped off, 3 = over four leaves wilted or dropped off, 4 = the whole plant died or all leaves dropped off. DI = [Σ(Number of diseased plants × corresponding level)/(Total number of surveyed plants × 4)] × 100; a higher DI indicates more serious infection. For the detection of defense-related marker genes, quantitative RT-PCR was conducted using total RNA samples from leaves and specific primers (Supplementary Table S1). At least two independent experiments were conducted with similar results.

### 3.12. RNA-Seq

Whole-plant tissue samples (excluding cotyledons) were collected 17 d after VIGS treatment and immediately flash-frozen in liquid nitrogen for storage. The samples were prepared with three biological replicates, and each replicate was pooled from six individual plants. Total RNA was isolated using TRIzol reagent (Invitrogen, Waltham, MA, USA). RNA purity and integrity were assessed using a NanoDrop 2000 spectrophotometer (NanoDrop Technologies, Wilmington, DE, USA) and a Bioanalyzer 2100 system (Agilent Technologies, Santa Clara, CA, USA). RNA contamination was assessed by 1.5% agarose gel electrophoresis. mRNA was purified from the total RNA using poly-T oligo-attached magnetic beads (Invitrogen). Sequencing libraries were prepared from purified mRNA using the VAHTS Universal V6 RNA-seq Library Kit for MGI (Vazyme) following the manufacturer’s recommendations with unique index codes. The library quantification and size were assessed using a Qubit 3.0 Fluorometer (Life Technologies, Carlsbad, CA, USA) and a Bioanalyzer 2100 system (Agilent Technologies). Subsequently, sequencing was performed on the MGI-SEQ 2000 platform (MGI Tech, Shenzhen, China) at Frasergen Bioinformatics (Wuhan, China). Low-quality reads, such as reads with adaptor sequences or reads with >5% N or >20% bases with quality < Q20 (percentage of sequences with sequencing error rates < 1%), were removed using Perl script. The clean data were mapped to the *G. hirsutum* ‘TM-1’ reference genome (https://www.cottongen.org/species/Gossypium_hirsutum/nbi-AD1_genome_v1.1 (accessed on 8 October 2020)) using HISAT2 [104]. Novel transcripts from the genome alignment were predicted using StringTie [105] and merged with the known transcripts from the ‘TM-1’ genome NAU-NBI_v1.1 as the merged transcriptome set. The reads were finally mapped to the merged transcriptome set using Bowtie2 [106], and the normalized expression level (fragments per kilobase of exon per million mapped fragments; FPKM) of each gene was quantified using RSEM [107].

### 3.13. DEGs and Functional Enrichment Analysis

Differentially expressed genes (DEGs) between sequencing sample groups were evaluated via DESeq2 [108]. The false discovery rate (FDR) was used to identify the threshold of the *p* value in multiple tests in order to compute the significance of the differences. DEGs were defined by the threshold of |log2(FoldChange)| > 1 and FDR-adjusted *p*-value (*p*_adj_) < 0.05. For quality control (QC), we validated the reliability of RNA-seq data via quantitative RT-PCR detection of 20 randomly selected up- and down-DEGs, obtaining a highly significant correlation (*R*^2^ = 0.98, *p* < 0.0001) between the results of RNA-seq and quantitative RT-PCR (Figure 5B,C).

The GO database (http://www.geneontology.org/ (accessed on 10 October 2020)) was used for GO enrichment analysis. KEGG pathway enrichment analysis was performed using KOBAS [109]. A hypergeometric test was implemented for the enrichment analyses, and a Benjamini–Hochberg adjusted FDR ≤ 0.05 was designated as statistically significant. The list of significantly enriched GO biological processes was subjected to analysis on the web server REVIGO (http://revigo.irb.hr/ (accessed on 7 June 2023)) [59], which resulted in a representative subset of the list by removing the redundant terms (default cutoff value of similarity: C = 0.7) and applying a stringent dispensability cutoff (<0.05). Subsequently, this representative subset of the enriched GO terms was visualized by plotting as described previously [110].

### 3.14. TF Family Enrichment Analysis

The presence of transcription factors (TFs) among the DEGs was predicted using Hidden Markov Model scan (hmmscan) [111] and the PlnTFDB database [112]. Then, a TF family enrichment analysis in the DEGs was conducted using a two-sided Fisher’s exact test to determine the *p*-value for statistical difference significance against the background TF database PlantTFDB [64].

### 3.15. KEGG Pathway Mapping

The KEGG pathways of plant–pathogen interactions (https://www.genome.jp/entry/map04626 (accessed on 12 October 2020)) and photosynthesis (https://www.genome.jp/entry/map00195 (accessed on 12 October 2020)) were annotated with the DEGs from *GhCNGC13*- and *32*-silenced plants versus mock control plants. The pathway was schematically depicted following the KEGG pathway maps. 

### 3.16. Statistical Analysis

Student’s *t*-tests were conducted using Microsoft Excel version 2311 (Microsoft Corporate, Redmond, WA, USA). A one-way analysis of variance (ANOVA) followed by Tukey’s multiple comparisons test was performed using GraphPad Prism 8.0.2 (GraphPad Software Inc., San Diego, CA, USA).

## 4. Conclusions

CNGCs have been extensively studied in *Arabidopsis thaliana* model plants in recent decades. However, little is known about the functional diversification of CNGCs in crop plants that are mostly polyploid species. In Upland cotton, GhCNGC13 and 32 are the closest homologs to AtCNGC2, which has been implicated in various signaling events, including Ca^2+^/CaM, cyclic nucleotide (cAMP and cGMP), phytohormone (i.e., IAA, SA, and JA), NO, CLV3/CLV1, and light signaling. Here, through VIGS experiments, we confirmed that silencing *GhCNGC13* and *32* resulted in plant growth suppression and autoimmune phenotypes (e.g., leaf necrosis or lesions, cell death, H_2_O_2_ accumulation, PR induction, SA and JA elevation) that are similar to the phenotypic effects of AtCNGC2 loss-of-function mutants. We also found that silencing *GhCNGC13* and *32* impaired SAM development and photosynthesis and raised ABA and GA_3_ as well as IAA. *GhCNGC13*- and *32*-silenced plants conferred an enhanced resistance to the airborne fungal pathogen *Botrytis cinerea*, but a compromised resistance to the soilborne fungus *Verticillium dahlia*. A large portion (6.6%) of expressed genes in the genome were differentially regulated in the silenced plants, which are enriched in functions predominately related to immunity and photosynthesis. Notably, most of the DEGs annotated in the plant–pathogen interaction pathway were upregulated, whereas all the DEGs in the photosynthesis pathway were downregulated. Among the DEGs, there was a group of TFs over-represented in the HSF, NAC, and WRKY families, as well as many members of the GhCNGC family, which may be indicative of a coordinated regulation. Altogether, these observations support the critical role of *GhCNGC13* and *32* in balancing plant growth and immunity, which may be associated with photosynthesis and/or an intricate regulatory signaling network that deserves further investigation. We postulated that *GhCNGC13* and *32* are key players in the “growth–defense tradeoff” of crops.

## Data Availability

The RNA sequencing data were deposited in the NCBI SRA database under the BioProject ID PRJNA1008288, accession numbers SRR25734064, SRR25734065, SRR25734066, SRR25734067, SRR25734068, and SRR25734069.

## Supplementary Materials

The supporting information can be downloaded at: https://www.mdpi.com/article/10.3390/ijms25010001/s1.
